# Posthospital Discharge Medical Care Costs and Family Burden Associated with Osteoporotic Fracture Patients in China from 2011 to 2013

**DOI:** 10.1155/2015/258089

**Published:** 2015-06-28

**Authors:** Zhao Xie, Russel Burge, Yicheng Yang, Fen Du, Tie Lu, Qiang Huang, Wenyu Ye, Weihua Xu

**Affiliations:** ^1^Southwest Hospital, Third Military Medical University, Chongqing 400038, China; ^2^Eli Lilly and Company, Indianapolis, IN 46285, USA; ^3^Lilly Suzhou Pharmaceutical Co., Ltd., Shanghai Branch, Shanghai 200001, China; ^4^Beijing Brainpower Pharma Consulting Co., Ltd., Beijing 100000, China; ^5^Beijing Chaoyang Hospital, Capital Medical University, Beijing 100026, China; ^6^Union Hospital, Tongji Medical College, Huazhong University of Science and Technology, Wuhan 430074, China

## Abstract

*Objectives*. This study collected and evaluated data on the costs of outpatient medical care and family burden associated with osteoporosis-related fracture rehabilitation following hospital discharge in China. *Materials and Methods*. Data were collected using a patient questionnaire from osteoporosis-related fracture patients (*N* = 123) who aged 50 years and older who were discharged between January 2011 and January 2013 from 3 large hospitals in China. The survey captured posthospital discharge direct medical costs, indirect medical costs, lost work time for caregivers, and patient ambulatory status. *Results*. Hip fracture was the most frequent fracture site (62.6%), followed by vertebral fracture (34.2%). The mean direct medical care costs per patient totaled 3,910¥, while mean indirect medical costs totaled 743¥. Lost work time for unpaid family caregivers was 16.4 days, resulting in an average lost income of 3,233¥. The average posthospital direct medical cost, indirect medical cost, and caregiver lost income associated with a fracture patient totaled 7,886¥. Patients' ambulatory status was negatively impacted following fracture. *Conclusions*. Significant time and cost of care are placed on patients and caregivers during rehabilitation after discharge for osteoporotic fracture. It is important to evaluate the role and responsibility for creating the growing and inequitable burden placed on patients and caregivers following osteoporotic fracture.

## 1. Introduction

Osteoporosis is a disease characterized by low bone mass and microarchitectural deterioration of bone tissue, leading to enhanced bone fragility and consequent increase in fracture risk [1]. Because osteoporosis itself has no symptoms, many patients are diagnosed only when a fragility fracture resulting from mechanical forces that would not ordinarily result in fracture has occurred [2, 3].

The prevalence of osteoporosis increases with age. In China, the prevalence of osteoporosis in the general population has been estimated at 7.0% [4], which accounts for 88.26 million osteoporotic patients having the disease and facing higher risk of fractures. The tremendous clinical consequences of osteoporosis-related fractures, and especially hip fractures, are significantly increased mortality and disability. Nearly one-quarter of osteoporosis patients die in the first year after fracture, with elderly men in all fracture groups having consistently higher standard mortality ratios (2.2–3.2) than elderly women (1.7–2.2) [5]. Furthermore, nearly 25% of patients refracture within 5 years, and excess mortality may be elevated for up to 10 years [6]. Hip fracture is the most severe type of fracture caused by osteoporosis [7] and normally requires surgical treatment and physical rehabilitation to help patients regain physical function and mobility.

Another significant consequence of osteoporosis-related fracture is the economic and social burden on the health care system and on society [8]. Osteoporosis-related fracture normally requires hospitalization care for surgery and complication management. The common comorbidities associated with elderly patients could significantly extend the length of hospitalization and increase hospitalization costs after surgery [9].

The overall prevalence of osteoporosis, as well as the clinical and economic burden, may grow substantially due to China's rapidly aging population. By 2030, the population aging 65 and older is expected to double to more than 238 million [11]. Conversely, the working population that aged 15 to 59 years totaled 937.27 million in 2012 and decreased by 3.45 million compared to 2011; thus, the proportion of working-age to total population in 2012 fell by 0.6% to 69.2% [10].

Estimates of the national total posthospital direct medical and nonmedical costs and indirect nonmedical costs are quite limited for China. Costs per hip fracture in China have been reported to range between 22,088¥ (US $3,600) and 34,202¥ (US $5,653), while average costs per vertebral fracture and nonhip/nonvertebral fracture were estimated to be 19,239¥ (US $3,175) and 22,034¥ (US $3,636), respectively [12–15]. The shares of the total direct medical costs due to hip, vertebral, and nonhip/nonvertebral fracture are not known for China, though in the United States these shares are 72%, 6%, and 22% [16], while in the European Union the corresponding shares are 54%, 5%, and 40% [17]. Very little is known about the share of the full cost burden attributable to indirect costs, though recent studies in Europe suggest that the share is nearly one-half [18, 19].

New research is needed on the full clinical and economic impact of osteoporosis in China to raise the disease awareness and to guide future health policy-making for reducing the potential increase of inequity and burden of osteoporosis in the country. The China public health insurance system now covers 95% of the population due to efforts to establish universal coverage under three primary government programs: Urban Employee Basic Medical Insurance (UEBMI), Urban Resident Basic Medical Insurance (URBMI), and New Rural Cooperative Medical Scheme (NRCMS). At this time, UEBMI covers inpatient costs and most outpatient costs, and URBMI and RNCMS only cover inpatient costs. However, there are limitations in the coverage of this insurance, potentially placing excess burden of costs and care on the patient and family.

Therefore, the objective of this study was to obtain and evaluate new data on the economic and social burden of osteoporotic fractures in China by conducting a survey of fracture patients between January 2011 and January 2013 of posthospital discharge direct and indirect medical care costs, indirect costs on families, and patients' disability status.

## 2. Materials and Methods

A retrospective survey was administered by doctors using a questionnaire completed by their patients and/or caregivers about postdischarge care. The questionnaire collected data on demographics; ambulatory status; direct medical care services and costs (outpatient services/visits (physician visits, emergency room visits); pharmaceutical drugs; rehabilitation care; nursing care; and devices and supplies); indirect medical services and costs (transportation, rehabilitation, home modifications, nutritional services, etc.); disability (before fracture and after fracture); and lost work time for caregivers. Caregivers were defined as family members of the patient for whom they provided care.

Potential patients for inclusion in the survey were identified after hospitalization for osteoporotic fracture using the following criteria:

1. Age of 50 years or above at hospital admission;
2. Confirmed diagnosis of osteoporosis by physician or diagnostic guidelines (bone mineral density *T*-score 2.5 standard deviations or more below the young adult peak mean);
3. Fracture that is not related to severe trauma (patients with fractures due to car accidents or other severe traumas were excluded);
4. Any significant comorbidities including stroke, dementia, and incurable cancers that have significant impact on health resource utilization were excluded;
5. Exclusion of patients with fractures of skull, facial, finger, and toe bones;
6. Discharge between January 1, 2011, and January 31, 2013.

The survey was conducted by a panel of 6 experienced physicians in the osteoporosis-related field in three Tier 3 comprehensive hospitals representing eastern, middle, and western China. The interviews were performed between November 15, 2012 and March 31, 2013.

### 2.1. Statistical Analysis

Descriptive statistical methods were used to produce a profile of patients' baseline characteristics, fracture sites, costs, and ambulatory status for osteoporotic fracture patients. McNemar's test was used to compare patient mobility status before fracture and after hospital discharge.

## 3. Results

### 3.1. Patient Demographics

The median number of days between fracture and survey interview was 149, with a minimum of 6 days and a maximum of 728 days. About 55% of responses were 3 to 6 months from fracture, and about 39% were between 6 and 12 months. A total of 189 patients met the inclusion criteria and were called by doctors; 172 patients answered the calls and responded for a response rate of 91.0%. A total of 123 questionnaires were completed, validated, and used for analysis for an effective rate of 71.5%.

The discharge distribution for the 123 patients across the three hospitals was Beijing hospital (42.3%), Wuhan hospital (24.4%), and the Chongqing hospital (33.3%) (Table 1). Patients were mostly female (64.2%), mean age was 71.3 years, and 30.1% were aged 80 or older (Table 1). Over 90% were residing in urban residences, and most were retired (71.6%) and living with their spouse (73.2%) (Table 1). While only 5% of patients were not insured, the majority of patients were insured by UEBMI (46.3%), URBMI (30.9%), and RNCMS (13.0%). Currently, UEBMI covers inpatient costs and most outpatient costs. However, URBMI and RNCMS only cover inpatient costs.

### 3.2. Fracture Types and Comorbidities

Among the 123 patients, all received fracture-related surgery, and most patients (91.1%) had sustained only one fracture, whereas 10 (8.1%) patients had sustained 2 fractures and only 1 (0.8%) patient had sustained 3 fractures (Table 2). Hip fracture was the most frequent fracture site (62.6%), followed by vertebral fracture (34.2%) with 29 lumbar vertebral and 13 thoracic vertebral fractures. The mean number of comorbidities was 2.3 per patient, and 27.6% of patients had more than three. The top five comorbid conditions included hypertension, rheumatoid arthritis, high cholesterol, cardiovascular disease, and chronic lung disease (Table 2).

### 3.3. Outpatient Visits

The average number of outpatient visits from discharge until interview was 1.7 per patient. While most patients (78.9%) had between 1 to 3 outpatient postdischarge visits, 4% of patients had more than 3 follow-up visits and 17.1% of patients had no follow-up visits.

### 3.4. Outpatient Direct Medical and Indirect Medical Care Costs and Family Burden

The costs of outpatient direct medical and indirect medical care are provided in Table 3. Direct medical costs (outpatient visits, pharmacy, device/supplies, rehabilitation, and nursing care) after hospital discharge resulted in an average cost of 3,910¥ per patient. Nursing care accounted for most of these direct costs (mean of 2,187¥), followed by outpatient visits (mean of 794¥). The total mean indirect medical costs per patient were 743¥ (nutrition, transportation, accommodation, and house modification).

Reported work time lost for caregivers to care for family members for recovery at home was the largest portion of nonhospital costs. The mean number of lost workdays was 16.4 days and varied widely between the three districts: It was the greatest in Wuhan (28.2 ± 41.0; 30 patients), followed by Chongqing (12.8 ± 23.7; 41 patients), and Beijing (12.4 ± 10.1; 52 patients) (Table 4). The mean regional daily income in 2011 for the three districts also varied widely: Beijing (301¥), Chongqing (157¥), and Wuhan (144¥). The mean caregiver lost income, therefore, was estimated by multiplying the mean regional daily income by the mean days of time lost for caregivers and was 2,007¥ in Chongqing, 4,061¥ in Wuhan, and 3,722¥ in Beijing. The overall weighted average caregiver lost income was 3,233¥ (i.e., weighted by sample sizes).

The overall estimate for total average nonhospital costs per patient was 7,886¥, which is the sum of nonhospital direct medical costs (3,910¥), indirect medical costs (743¥), and average caregiver lost income (3,233¥) for the 123 patients in the sample.

### 3.5. Mobility

Table 5 presents mobility of osteoporotic patients before fracture and after discharge from hospital. The vast majority of patients (87%) could walk unaided before their fracture, but this was reduced after fracture (64%, *P* < 0.001). Of the 123 patients, 12% were able to walk with assistance before their fracture, but after fracture 27% required assistance with walking (*P* < 0.001). Only 1 patient was confined to a wheelchair before the fracture, but, after fracture, there were 7 patients confined to wheelchairs (*P* = 0.014), whereas none were confined to the house (bedridden) due to immobility before fracture, and 4 patients were confined to the house after fracture.

## 4. Discussion

The burden of osteoporosis is expected to increase in China due to the rapidly aging population. Some limited data exist regarding inpatient costs of fractures in China [12, 20–23]. However, the full burden due to fractures is unknown. To gain a better understanding on the economic and societal burden of osteoporotic fractures, we conducted a survey to collect direct medical costs, indirect medical costs, and family burden of lost work time following hospital discharge for fracture patients. The results from our survey indicated that within direct medical costs, the highest cost is nursing care, which is often paid for by the patient. Also, caregivers lost, on average, 33.2 working days (maximum 395 days), daily median incomes ranged from 144¥ to 301¥, and total lost income substantially exceeded the combined cost of outpatient direct and indirect medical costs. In addition, patient mobility postacute care was significantly reduced regarding the patients' ability to walk unaided, freedom from wheelchair, or not being bedridden, indicating greater disability and subsequent need for caregiver assistance as well as home modifications to prevent slippage and assist mobility.

The burden on families appears to be substantial from these findings. Possible reasons for this high family burden include a lack of formal rehabilitation facilities [24], current health insurance plans to cover outpatient rehabilitation costs, and patient preferences to home care, cultural expectations, and honoring older family members by providing home care. Lost income for caregivers is anticipated to increase due to the overall rising incomes and to the one-child per family policy, which reduces the number of children that can share the caregiver burden.

Our study provides some insights on costs following discharge from the hospital. We found that families are bearing a substantial burden which consists of nursing services, devices and supplies, transportation, and nutrition. However, the amount of work time and income lost for caregivers was more than direct and indirect medical care costs combined. The burden may fall unevenly across families, thus resulting in extreme hardships and social inequities. The study surveys recent fracture patients and is comprehensive in posthospital direct and indirect medical costs, as well as nonmedical costs. Some important limitations are the inclusion of geographic areas that may not be fully representative of all China, a small sample size relative to the national population, and patients' memory recall that may be diminished or less accurate, especially for those with a longer gap between the hospital discharge and interview date. In addition, this analysis was not able to validate the medical encounters or the costs. Also, we could not ascertain causality of osteoporosis and hip fractures, which mostly occur following a fall [25]. As many of our responses were obtained within 3 to 6 months after discharge, we may have underestimated the full impact for a full year of costs and burdens following hospital discharge or overestimated indirect costs since these values are not verified.

Nevertheless, this study provides important new information and insights about the additional burden fracture and potential social and family inequities after hospital discharge. This study may serve as an important guide for a larger, nationally based survey.

## 5. Conclusions

The expected growth in osteoporosis and osteoporotic fractures will impose a growing clinical, economic, and humanistic burden on many developing and nondeveloping countries in the world. However, China, a country with recently expanding economic advantages and unique population policy, has a far more complex situation. As the majority of postfracture osteoporotic patients lack health insurance coverage for rehabilitation care and also relatively few community rehabilitation facilities, rehabilitation is mostly conducted at home where the typical family has fewer caregivers than caregiving families in other countries. The current family burden in China may grow even faster as the shifts in demographics move closer toward an increasing older population and declining working-age population over the coming decades. With the rising incomes, even larger indirect costs may be incurred due to lost workdays by family members/caregivers. Further research is needed to better quantify the full clinical, economic, and social burden and to guide resource allocations to improve outcomes and reduce inequities across China.

## Figures and Tables

**Table 1:** Patient demographics.

Sample size	*N* = 123
Hospital location, *n* (%)	
Beijing	52 (42.3)
Wuhan	30 (24.4)
Chongqing	41 (33.3)

Gender, *n* (%)	
Female	79 (64.2)
Male	44 (35.8)

Age, mean (SD)	71.3 (11.4)
Age group, *n* (%)	
50–59	26 (21.1)
60–69	24 (19.5)
70–79	36 (29.3)
80+	37 (30.1)

Health insurance type, *n* (%)	
UEBMI	57 (46.3)
URBMI	38 (30.9)
RNCMS	16 (13.0)
Not insured	6 (4.9)
Free medical care	3 (2.5)
Commercial insurance	3 (2.4)

Marital status, *n* (%)	
Living with spouse	90 (73.2)
Divorce	1 (0.8)
Widowed	32 (26.0)

Current residence, *n* (%)	
Urban	111 (90.2)
Rural	12 (9.8)

Work status, *n* (%)	
Retired	88 (71.6)
On-the-job	9 (7.3)
Others	26 (21.1)

RNCMS = Rural New Cooperative Medical System; SD = standard deviation; UEBMI = Urban Employee Basic Medical Insurance; URBMI = Urban Resident Basic Medical Insurance.

**Table 2:** Baseline fractures and comorbidities.

	*N* = 123
Fractures, *n* (%)	
1	112 (91.1)
2	10 (8.1)
3	1 (0.8)

Fracture type, *n* (%)	
Vertebral	42 (34.2)
Thoracic vertebra	13
Lumbar vertebra	29
Hip	77 (62.6)
Nonhip/nonvertebral	3 (2.4)
Multiple fracture sites	1 (0.8)

Comorbidities	
Mean (SD)	2.3 (1.7)
0	24 (19.5)
1~3	65 (52.9)
>3	34 (27.6)

Common comorbidities, *n* (%)	
Hypertension	75 (61.0)
Rheumatoid arthritis	51 (41.5)
High cholesterol	43 (35.0)
Cardiovascular disease	42 (34.2)
Chronic lung disease	33 (26.8)

SD: standard deviation.

**Table 3:** Outpatient direct medical and indirect medical care costs^*∗*^ (¥).

	Cost from discharge to interview	
	Mean ± SD	Median
Outpatient direct medical care		
Postdischarge outpatient visits	794 ± 634	650
Pharmacy	333 ± 830	0
Device/supplies	563 ± 705	300
Rehabilitation	33 ± 219	0
Nursing care	2,187 ± 4,424	1,200

Total mean outpatient direct medical costs	3,910	2,150

Indirect medical costs		
Nutrition	350 ± 1,083	0
Transportation	221 ± 242	200
Accommodation	132 ± 244	0
House modification (e.g., slip proof)	40 ± 105	0

Total mean outpatient indirect medical costs	743	200

^*∗*^Calculated cost per patient from discharge to interview.

**Table 4:** Caregiver lost work time (days) and lost income (¥).

	Total (*n* = 123)	Beijing (*n* = 52)	Wuhan (*n* = 30)	Chongqing (*n* = 41)
Required a caregiver with lost work time, *n* (%)	72 (61)	41 (80)	15 (53)	16 (41)
Caregiver lost workdays				
Mean ± SD	16.4 ± 26.0	12.4 ± 10.1	28.2 ± 41.0	12.8 ± 23.7
Median	9.0	13.0	3.5	0
Range (minimum, maximum)	(0, 173)	(0, 44)	(0, 173)	(0, 105)
Mean regional daily income (2011, ¥)^*∗*^		301	144	157
Caregiver lost income, mean ± SD (¥)	3,233 ± 4,184	3,722 ± 3,054	4,061 ± 5,899	2,007 ± 3,728
Caregiver lost income, median (¥)	2,408	3,913	504	0

^*∗*^Source: National Bureau of Statistics of China, 2013 [10].

**Table 5:** Mobility status, prefracture, and posthospital discharge.

*N* = 123	Before fracture *n* (%)	After discharge *n* (%)	*P* value (McNemar's test)
Ambulatory status			

Walked without any help	107 (87)	79 (64)	<0.001
Walked with aid	15 (12)	33 (27)	<0.001
Could not walk, confined to house	0	4 (3)	—
Could not walk, use of wheelchair	1 (1)	7 (6)	0.014

Not ambulatory	16 (13)	44 (36)	<0.001
